# Wellbeing and Happiness and Their Association With Working Conditions at Sea: A Cross-sectional Study Among the Global Workforce of Seafarers

**DOI:** 10.1177/00469580241256349

**Published:** 2024-05-30

**Authors:** Rebecca Hayes-Mejia, Martin Stafström

**Affiliations:** 1 Lund University, Sweden; 2 Marine Benefits AS, Bergen, Norway

**Keywords:** seafarers, wellbeing, happiness, cross-sectional survey data, global workforce

## Abstract

The aim of this study was to investigate whether seafarers’ self-reported work experiences were associated with wellbeing and happiness while onboard. The study also examined which indicators of the work experiences had an effect in what direction. We analyzed the survey responses from 13 008 seafarers onboard, from 154 different nationalities, serving in 44 different international shipping companies. The outcome measures were wellbeing and happiness, and the exposure variables were work environment factors: satisfaction, expectations, ideal, skills and training, challenges, and workload. General psychosocial work environment onboard and socioeconomic independent variables were also included. We conducted different logistic regression analyses, and presented the results as odds ratios (OR) and 95% confidence intervals (CIs). The study found that most seafarers reported high levels of wellbeing and happiness and that these were significantly associated to the work environment factors, except for workload. A stratified analysis, showed that workload modified the effect of the other work environment factors. The study found that there were independently significant associations between work related factors and wellbeing and happiness among seafarers at sea. The findings suggest that a greater emphasis on these outcomes could have a positive impact both on crew retention and safety at sea.

**What do we already know about this topic?**
That wellbeing and happiness affects life at sea in international shipping, both in terms of crew retainment and safety.
**How does your research contribute to the field?**
By the study’s uniquely large sample it confirms small sample study findings, as well as establishing that wellbeing and happiness is associated with the work environment onboard.
**What are your research’s implications toward theory, practice, or policy?**
The findings suggest that if wellbeing and happiness would be emphasized more by shipping companies when managing seafarers, it could have a positive impact both on crew retention and safety at sea.

## Introduction

The wellbeing and happiness of seafarers are fundamental to safe and efficient shipping. The 1,8 million seafarers serving in the world’s commercial shipping industry play a crucial role in the global supply chain. They serve on more than 75 000 vessels around the globe.^1^ It is well established that seafaring is a stressful and high risk occupation, not only when on active duty, but also when off-duty.^2^ Seafaring is one of the most dangerous occupations in the world, which exposes mariners to chemical, biological, and ergonomic hazards with risks of occupational accidents, injuries, and diseases.^3^ Moreover, the social and psychological aspects of life onboard is challenging and observed to have great negative impacts on the mental health among seafarers.^4-6^ The most important challenges observed are shift work, noisy environment, harsh weather conditions, loneliness onboard, limited shore leave and recreational activities, long separation from families, fatigue, and sleep deprivation.^6,7^ Other factors contributing to mental health issues among seafarers are lack of adequate training, job satisfaction, and social support, but also working under time pressure and an uncaring work environment.^5,8^

Seafarers’ mental health and wellbeing have over the last few years become an increasing concern in the maritime industry. In their study, Sampson and Ellis^9^ concluded that the wellbeing and mental health of seafarers were of great concern to maritime charities, trade unions and employer associations but less so among employers in the industry. Their research also found that addressing employment terms and conditions, physical health, communication structures, and recreational facilities onboard, were effective ways to improve happiness and wellbeing among seafarers, compared to the reactive approaches, such counseling services and self-help, often used today. A recent study found that stress, anxiety, and depression among seafarers were significantly affected by the work environment onboard as well as the communication from employers.^10^ However, despite the many hardships, many seafarers do find work onboard engaging, satisfying, and are highly committed to their workplace and profession.^11^ In their study, Nielsen et al,^12^ observed that physical and psychosocial conditions at work were found to be correlated to job satisfaction and team cohesion, which in turn are strong indicators of work-related wellbeing.

### Work and Wellbeing

Work has a considerable impact on health and wellbeing. When a person feels satisfied, motivated, efficient and confident that everything is under control, there is a balance between that person’s resources and challenges.^13,14^ This positive state of work engagement and satisfaction is observed to be associated with both psychological and physical determinants of health such as wellbeing, happiness, stress and has also shown to lower and blood pressure.^15^ A supportive and engaging workplace has similarly shown to be an important factor contributing to job satisfaction and engagement with many positive health effects, such as reduced stress levels, and improved mental wellbeing and happiness.^16^ Other positive health effects that have been described in the literature are increased physical activity, enhanced ergonomics, lower absenteeism, stronger social connections, increased safety awareness and reduced risks of occupational hazards.^17,18^ In contrast, a less optimal workplace can impact health negatively, leading to a wide range of health problems such as occupational injuries, accidents, musculoskeletal conditions from poor ergonomics, stress, burnout,^19^ anxiety,^6^ depression, sedentary behaviors, sleep disorders, and fatigue.^20^

### Mental Health, Happiness and Wellbeing

Health is a broad and complex term. It includes physical, mental, and social wellbeing, and is “not merely the absence of disease or infirmity,” as defined by the World Health Organization.^21^ This deficit-oriented definition of health has moved toward one of positive psychological health and wellbeing.^22^ The WHO defines mental health as “a state of wellbeing in which the individual realizes his or her own abilities, can cope with normal stressors of life, can work productively and fruitfully, and is able to make a contribution to his or her community.”^23^ Further, mental health is fundamental for people to fulfill their lives and a major concern for both the individual and the community in which he or she exists. Dodge et al^24^ defines wellbeing as the balance point between an individuals’ resources and challenges. In essence, the state of wellbeing is reached when an individual has the psychological, social, spiritual, and physical means necessary to meet a specific challenge. In other words, if a person faces more challenges than he has resources, the wellbeing levels will reduce, and vice versa.

Wellbeing and happiness are subjective experiences and evaluations of a person’s own life, regardless of how others see it. Wellbeing and happiness are related but represent different aspects of the individual’s overall state.^25,26^ To achieve these experiences, one needs to feel accomplishment, have some control over one’s life and living conditions as well as a sense of purpose and meaning in life.^27^ It is generally agreed that wellbeing is broad, multidimensional and expands beyond merely feeling happy or being satisfied with life.^28^

The concepts of happiness and wellbeing draw upon various theories where the most prominent ones are Hedonism and Eudaimonism.^29,30^ The hedonic theory focuses on happiness as the central component of wellbeing and suggests that wellbeing is achieved through the search of pleasure and avoiding pain. Eudaimonism pursues meaning, self-actualization and growth and where wellbeing is the result of living in agreement with one’s values and virtues.^29^ Happiness is generally referred to as a positive emotional state characterized by feelings of joy, contentment, and satisfaction. Wellbeing is a holistic concept that includes happiness but assesses broader dimensions of life.^31^ In Labonté’s^32^ model, wellbeing is central and supported by three overlapping factors; physical health, mental wellbeing, and social connections and support. It is important to note that mental health and wellbeing are also significantly affected by a person’s individual personality traits,^33,34^ as well as social capital and social support.^35^ Among seafarers onboard, social support has shown to be a critical mitigating factor for wellbeing.^8^

From the occupational perspective of seafarers, wellbeing and happiness are interesting from mainly two points of view. First, it addresses the overall state among the global workforce of seafarers, indicating to what extent their general state of mind is as opposed to studies of adverse outcomes of mental health, which rather focus on the prevalence of pathological diagnoses rather than the overall mood. If one, for example, would be interested in analyzing retention rates within the industry, then wellbeing and happiness is likely a better indicator at an initial stage in such an analysis than psychiatric diagnoses. A second reason for studying wellbeing and happiness from a seafarer perspective is the link between these two states and safety. Studies have found that an important condition to establish a safety culture onboard a ship is the wellbeing of the crew.^12,36^ However, in order to understand the link between the working conditions, further research is needed as the previous studies have, hitherto, either been small in terms of population size or done within national samples of seafarers, somewhat disregarding the global recruitment of vessel crews.

### Aim

The aim of this study was to investigate whether seafarers’ self-reported work experiences were associated with wellbeing and happiness while onboard. The study also examined which indicators of the work experiences had an effect in what direction.

## Methods

### Population and Data Sampling

This cross-sectional study was carried out in 2022 among international seafarers from 44 different shipping companies. The data collected on wellbeing and happiness were part of a larger study by a collaboration between Lund University and Marine Benefits, a Norwegian insurance company providing health insurance for crews in international shipping.^10^ In total the survey received responses from 24 662 seafarers that were both at home and onboard at the time. This study is limited to the 13 008 seafarers who were onboard ship at the time of responding to the survey, as they were all exposed to the same environment, for example, the work environment onboard. The work environment onboard is essential to this study as we are specifically interested in how it affects seafarers’ wellbeing and happiness.

The questionnaires were sent out electronically through various HR departments and manning agencies. The seafarers received a link or a QR code to the survey which they answered anonymously. The full survey included 41 questions with its focus on mental health and wellbeing through various psychometric scales, which all were independently validated.^10^ All seafarers were asked to give their informed consent before completing the questionnaire. The study was given ethical approval by the Swedish Research Council (decision 2022-00444-01).

### Measurements

This study has its focus on wellbeing and happiness and how these relate to seafarers’ subjective work experience while onboard their vessels. The outcome variables were wellbeing and happiness.

Wellbeing was measured through the WHO-5 index.^37,38^

The scale includes five items, each with scores ranging from 0 to 5. The total score of 25 represents the best imaginable wellbeing, and 0 represents the worst imaginable wellbeing. A score below 13 indicates poor wellbeing. The raw sore is multiplied by 4 to give the final score. The scale indicated a good internal reliability with Cronbach’s alpha of .79.

Happiness was measured through the Subjective Happiness Scale (SHS).^39^ The scale includes four items ranging from 1 to 7 points each. The scale is scored by computing the mean of the 4 items. The coding of the fourth item is reversed. The Scale showed a reliable internal reliability with a Cronbach’s alpha of .77. Higher scores reflect greater happiness.

Three categories of independent variables were included in the study. First, the main exposure variables were related to the seafarers’ subjective work experience onboard in relation to satisfaction, expectations, ideal, skills and training, challenges, and workload (See Table 1). The respondents were asked, on a scale of 0 to 10,: How satisfied are you with your current work? How well does your current work meet your expectations? How close is your current work to the ideal one? How relevant is your skillset to your work? How do you find your work in terms of challenges? How is your workload on an average day? (where 0 represents “not at all or low” and 10 “very much or very high”). These variables were trichotomized based on the mean and ±1 standard deviation, into the categories “Low,” “Medium,” “High.”

**Table 1.** Prevalence of Wellbeing, Happiness, Work Experience and Psychosocial Work Environment Among Seafarers at Sea (n = 13 008).

		Total	
Outcome variable	Category	n	%
Wellbeing	All well	10 047	77.24
Happiness	Happy	9941	81.86
Main exposure variables			
Satisfaction	Low	1574	16.08
	Medium	5316	54.31
	High	2898	29.61
Expectations	Low	1216	12.42
	Medium	6372	65.10
	High	2.2	22.48
Ideal	Low	1617	16.52
	Medium	6435	65.74
	High	1736	17.74
Training/Skills	Low	931	9.51
	Medium	6371	65.09
	High	2486	25.40
Challenges	Low	1172	11.97
	Medium	6816	69.64
	High	1,8	18.39
Workload	Low	1854	18.94
	Medium	6934	70.84
	High	1000	10.22
Psychosocial work environment			
I feel lonely	At no time	3601	36.23
	Some of the time	4515	45.42
	Less than half of the time	475	4.78
	More than half of the time	471	4.74
	Most of the time	491	4.94
	All of the time	387	3.89
I have at least one co-worker to talk to	At no time	1,52	15.29
	Some of the time	2644	26.60
	Less than half of the time	371	3.73
	More than half of the time	1509	15.18
	Most of the time	537	5.40
	All of the time	3359	33.79
I feel bullied	At no time	7576	76.22
	Some of the time	1598	16.08
	Less than half of the time	249	2.51
	More than half of the time	168	1.69
	Most of the time	173	1.74
	All of the time	176	1.77
I feel discriminated against	At no time	7392	74.37
	Some of the time	1792	18.03
	Less than half of the time	244	2.45
	More than half of the time	162	1.63
	Most of the time	186	1.87
	All of the time	164	1.65
We have a lot of group activities while onboard	At no time	1,45	14.59
	Some of the time	3577	35.99
	Less than half of the time	691	6.95
	More than half of the time	1986	19.98
	Most of the time	1089	10.96
	All of the time	1147	11.54
I get enough sleep	At no time	567	5.70
	Some of the time	2,31	23.24
	Less than half of the time	596	6.00
	More than half of the time	2807	28.24
	Most of the time	930	9.36
	All of the time	2,73	27.46

The second category of independent measurements assessed the general psychosocial work environment onboard. These were made up out of six questions around loneliness, having someone to talk to, feeling bullied, discriminated, group activities, sleep, and time onboard (See Table 1).

Variables indicating socioeconomic status were the third category of independent variables. These included sex, age, rank, and nationality. Age was categorized into groups. The first group ranged between 18 and 30 years, thereafter into 10-year intervals. Rank was the self-reported onboard placement, for the contract period. The ranks were then divided into 12 different categories.

The top 10 most common nationalities were coded into National Origin consisting of 10 different continental/regional categories: EU, non-EU Europe, North Africa, Sub-Saharan Africa, The Middle East/Gulf States and Central Asia, Sub-Continental Asia, East Asia, Oceania, North America, Latin America and, finally, the Caribbean, except for the 4 most common nationalities of Filipino, Indians, Russians, and Ukrainians.

### Statistical Analysis

All confidence intervals (CI) and odds ratios (OR) were computed using STATA 17. We first presented the prevalence of the outcome variables; wellbeing and happiness, main exposure variables; work experience and psychosocial work environment. Secondly, we applied a bivariate logistic regression analysis presented as bivariate odds ratios (OR) and 95% confidence intervals (CI). Thereafter, we conducted multivariate regression analyses for both outcome variables, wellbeing, and happiness respectively, presented as odds ratios (OR) and 95% confidence intervals (CIs). These were done in 3 steps. The first step only included the main exposure variables. In the second step we included socioeconomic factors, and in the third and final step we included both socioeconomic factors and the psychosocial environment factors. Lastly, we conducted a stratified multivariate regression analysis to further investigate and test for confounding effects, presented as odds ratios (OR) and 95% confidence intervals (CIs).

## Results

A total of 13 008 international seafarers participated in the study, all while sailing. Most seafarers reported high levels of wellbeing and happiness onboard, 77.2% and 81.7% respectively (see Table 1). As the main exposure variables were categorized using means and standard deviations, medium levels were more prevalent. A higher prevalence of seafarers reported job satisfaction, expectations, ideal workplace, training/skills, and challenges as high, compared to low. The opposite was observed for workload, where more seafarers rated workload as low.

Regarding the psychosocial environment, about a fifth reported feeling lonely, about a quarter bullied or discriminated against, and two-thirds reported not getting enough sleep. In addition, a majority reported that they did not have at least 1 co-worker to talk to, and 3 quarters did not have a lot of group activities while onboard.

In the dataset, presented in Table 2, seafarers from more than 100 different nationalities responded, the 3 most common being Filipino (42.4%), Indian (28.2%), and Ukrainian (5.8%), and Russian (2.87%), other nationalities were sorted into geographical regions. The sample was heavily biased toward being male (98.5%). Age was normally distributed, with the largest group being those 31 to 40 years of age (36%). According to rank, a third (32.4%) were officers, and two-thirds (67.6%) were ratings or other non-officers, with the remaining being undefined.

**Table 2.** Demographic Information of the Survey Participants (n = 13008).

Variable	Category	N	%
Sex	Female	191	1.47
	Male	12 817	98.53
Age	18-30	3579	27.51
	31-40	4687	36.03
	41-50	3131	24.07
	51-60	1396	10.73
	61+	215	1.65
Rank	Master/captain	992	7.85
	Chief Officer/deck manager	713	5.64
	Deck Officer/Deck Operations	1,61	12.74
	Deck Rating/Deck Support/Bosun	2889	22.87
	Chief Engineer/Engine Manager	778	6.16
	Engine Officer/Engine Operations	1711	13.54
	Engine Rating/Engine Support	1553	12.29
	Chief Cook/Catering Operations	529	4.19
	Galley staff/Catering Support	485	3.84
	Cadet/Trainee	416	3.29
	Painter	12	0.09
	Welder	210	1.66
	Other professions	736	5.83
Rank	Officer	4093	32.40
	Rating etc	8541	67.60
National origin	EU/EES	1333	10.25
	Europe—not EU	125	0.96
	North Africa	29	0.22
	Sub-Saharan Africa	141	1.08
	Middle East, Gulf States and Central Asia	59	0.45
	Eastern Asia, except Filipino	711	5.47
	Oceania	8	0.06
	North America	7	0.05
	Latin America	69	0.53
	Caribbean	18	0.14
	Sub-Continental Asia, not India	192	1.48
	Russian	373	2.87
	Indian	3671	28.22
	Filipino	5517	42.41
	Ukrainian	755	5.80
Time at sea	Less than 1 month	1372	10.86
	1 month	1329	10.52
	2 months	1714	13.57
	3 months	1,98	15.67
	4 months	1661	13.15
	5 months	1,37	10.84
	6 months	1152	9.12
	7 months	795	6.29
	8 months	556	4.40
	9 months	430	3.40
	10 months	160	1.27
	11 months	65	0.51
	12 months or more	50	0.40

Table 3 presents a bivariate logistic regression analysis. Each independent variable was analyzed separately against the two outcome variables wellbeing and happiness. All work experience variables; satisfaction, expectations, ideal, training and skills and challenges, except for workload were significantly associated with both wellbeing and happiness. Satisfaction, expectations, and ideal workplace had the strongest associations with both wellbeing and happiness, with ORs ranging between 3.2 and 4.5 with 95% CI. All the work experience variables had stronger associations to happiness compared to wellbeing. Workload was not found to be significantly associated to either wellbeing or happiness.

**Table 3.** Bivariate Logistic Regression Analysis—Subjective Work Experience Variables and the Odds for Wellbeing and Happiness Presented as Odds Ratios (OR) and 95% Confidence Intervals (CI) Among International Seafarers While Onboard.

	WHO-5			Happiness		
Variable	OR	95% CI		OR	95% CI	
Satisfaction	3.2	2.92	3.42	3.9	3.56	4.27
Expectations	3.9	3.56	4.28	4.5	4.09	5.04
Ideal	4.0	3.62	4.35	4.3	3.85	4.71
Training/Skills	2.3	2.07	2.46	2.8	2.51	3.06
Challenges	2.1	1.87	2.23	2.5	2.25	2.76
Workload	0.93	0.85	1.01	1.1	0.99	1.2

The multivariate logistic regression indicated significant associations between work satisfaction, expectations and ideal workplace and wellbeing in all three models (see Table 4). However, satisfaction and expectations and their association with wellbeing in model 3 were seen to be confounded by the psychosocial environmental factors. Training/skills was significantly associated with wellbeing in models 1 and 2 (OR 1.3 and 95% CI 1.03-1.60 and 1.04-1.64), but not in model 3, which was confounded by the psychosocial environmental factors. Medium challenges were observed to be significantly associated with wellbeing in all three models with ORs of 1.4 each and a 95% CI 1.14 to 1.63 in model 3. High challenges were in contrast not found to be associated with wellbeing in model 2 and 3. Workload was on the other hand found to be significantly negatively associated with wellbeing, in all three models. This means that the odds of wellbeing were reduced with increased workload.

**Table 4.** Multivariate Regression Analysis—Subjective Work Experience and Wellbeing (WHO-5) Presented as Odds Ratios (ORs) and 95% Confidence Intervals (CIs) Among International Seafarers Onboard.

		Model 1			Model 2			Model 3		
		OR	95% CI		OR	95% CI		OR	95% CI	
Satisfaction	Low									
	Medium	2.4	2.03	2.86	2.3	1.96	2.78	2.0	1.71	2.45
	High	2.5	1.96	3.09	2.3	1.81	2.87	1.9	1.52	2.45
Expectations	Low									
	Medium	1.7	1.40	2.09	1.8	1.45	2.18	1.6	1.25	1.92
	High	2.3	1.73	3.12	2.6	1.94	3.52	2.1	1.54	2.85
Ideal	Low									
	Medium	2.2	1.85	2.57	2.0	1.70	2.39	2.0	1.66	2.36
	High	3.1	2.35	4.04	2.8	2.08	3.64	2.6	1.94	3.44
Training/Skills	Low									
	Medium	1.2	0.99	1.43	1.2	1.02	1.47	1.1	0.93	1.36
	High	1.3	1.03	1.60	1.3	1.04	1.64	1.2	0.91	1.45
Challenges	Low									
	Medium	1.4	1.20	1.71	1.4	1.14	1.62	1.4	1.14	1.63
	High	1.3	1.01	1.58	1.2	0.95	1.51	1.2	0.97	1.55
Workload	Low									
	Medium	0.7	0.61	0.81	0.7	0.57	0.77	0.7	0.63	0.86
	High	0.5	0.41	0.63	0.5	0.40	0.61	0.6	0.49	0.76

*Note.* Model 1: unadjusted. Model 2: Adjusted for socioeconomic factors (sex, age, rank, and national origin). Model 3: Adjusted for socioeconomic factors (sex, age, rank, and national origin) and psychosocial environmental factors (loneliness, bullied, co-worker to talk to, felling discriminated, group activities, sleep and time onboard).

The multivariate regression analyses with happiness as the outcome (Table 5) indicated that the exposure variables satisfaction, expectations, ideal workplace, training, and challenges were all independently and significantly associated with happiness in all three models. Confounding effects were however found both between satisfaction and expectations in their association to happiness when adding the psychosocial work environment factors in model 3. High workload was negatively associated with happiness in model 1 and 2 with OR 0.7 (95% CI 0.52-0.83) and OR 0.6 (95% CI 0.50-0.81) respectively. In the third model, adjusted for both socioeconomic- and psychosocial environmental factors, no significant association was observed.

**Table 5.** Multivariate Regression Analysis—Subjective Work Experience and Happiness Presented as Odds Ratios (ORs) and 95% Confidence Intervals (CIs) Among International Seafarers Onboard.

		Model 1			Model 2			Model 3		
		OR	95% CI		OR	95% CI		OR	95% CI	
Satisfaction	Low									
	Medium	2.4	1.99	2.87	2.4	1.97	2.84	2.1	1.72	2.53
	High	3.5	2.71	4.60	3.4	2.62	4.51	3.0	2.27	3.97
Expectations	Low									
	Medium	1.5	1.24	1.89	1.5	1.23	1.89	1.3	1.04	1.62
	High	2.2	1.52	3.07	2.2	1.56	3.15	1.8	1.22	2.51
Ideal	Low									
	Medium	1.7	1.46	2.10	1.7	1.43	2.07	1.7	1.39	2.04
	High	2.2	1.57	3.05	2.1	1.52	3.00	2.0	1.45	2.88
Training/Skills	Low									
	Medium	1.4	1.13	1.63	1.4	1.13	1.64	1.3	1.06	1.56
	High	1.5	1.19	1.91	1.5	1.18	1.91	1.4	1.07	1.76
Challenges	Low									
	Medium	1.5	1.23	1.77	1.5	1.23	1.77	1.5	1.20	1.75
	High	1.4	1.10	1.83	1.4	1.11	1.85	1.5	1.14	1.93
Workload	Low									
	Medium	0.9	0.74	1.02	0.8	0.72	0.99	0.9	0.79	1.10
	High	0.7	0.52	0.83	0.6	0.50	0.81	0.8	0.61	1.00

*Note.* Model 1: unadjusted. Model 2: Adjusted for socioeconomic factors (sex, age, rank, and national origin). Model 3: Adjusted for socioeconomic factors (sex, age, rank, and national origin) and psychosocial environmental factors (loneliness, bullied, co-worker to talk to, felling discriminated, group activities, sleep and time onboard).

Table 6 presents a stratified multivariate regression analysis of wellbeing, happiness, and work experience, stratified by workload. The analysis indicated that the level of workload modifies the association between work experience and the outcome of wellbeing and happiness. Medium workload was observed to be most associated with both wellbeing and happiness. However, medium workload and training were not associated with wellbeing while they were significantly associated with happiness (OR 1.4 95% CI 1.4-1.99). Medium levels of challenges were significantly associated to both wellbeing and happiness at medium workload, while high challenges were not. Hence workload is observed to have a threshold effect toward both wellbeing and happiness.

**Table 6.** Stratified Multivariate Regression Analysis—Subjective Work Experience, Wellbeing and Happiness Stratified by Workload Presented as Odds Ratios (ORs) and 95% Confidence Intervals (CIs) Among International Seafarers Onboard.

		Low workload			Medium workload			High workload		
WHO-5		OR	95% CI		OR	95% CI		OR	95% CI	
Satisfaction	Low									
	Medium	2.0	1.29	3.03	1.9	1.56	2.40	1.2	0.61	2.23
	High	2.8	1.58	4.94	1.7	1.28	2.28	1.3	0.58	2.71
Expectations	Low									
	Medium	0.9	0.58	1.46	1.7	1.29	2.19	1.3	0.62	2.53
	High	1.4	0.66	2.81	1.9	1.28	2.73	1.4	0.57	3.44
Ideal	Low									
	Medium	2.0	1.34	2.91	1.9	1.57	2.40	2.4	1.32	4.24
	High	2.1	1.04	4.13	2.9	2.05	4.20	3.2	1.50	6.89
Training/Skills	Low									
	Medium	1.1	0.73	1.52	1.0	0.80	1.31	1.7	0.82	3.5
	High	1.0	0.62	1.60	1.0	0.72	1.32	2.0	0.95	4.36
Challenges	Low									
	Medium	1.3	0.98	1.78	1.5	1.13	1.87	1.7	0.81	3.46
	High	1.0	0.57	1.64	1.3	0.93	1.77	1.9	0.89	3.88
		Low workload			Medium workload			High workload		
Happiness		OR	95% CI		OR	95% CI		OR	95% CI	
Satisfaction	Low									
	Medium	1.6	1.04	2.49	2.0	1.56	2.46	3.2	1.55	6.46
	High	3.7	1.94	7.09	2.8	1.98	3.89	2.9	1.27	6.62
Expectations	Low									
	Medium	1.2	0.75	1.88	1.5	1.12	1.92	0.4	0.20	0.96
	High	1.2	0.53	2.54	1.9	1.20	2.93	0.9	0.32	2.75
Ideal	Low									
	Medium	2.0	1.35	3.00	1.4	1.14	1.81	2.5	1.33	4.85
	High	1.9	0.90	4.10	1.9	1.20	2.89	3.2	1.25	7.93
Training/Skills	Low									
	Medium	1.1	0.79	1.66	1.4	1.06	1.72	1.4	0.70	2.70
	High	1.1	0.68	1.84	1.4	1.05	1.99	1.5	0.72	3.08
Challenges	Low									
	Medium	1.5	1.08	1.99	1.4	1.10	1.84	1.6	0.80	3.20
	High	1.6	0.88	2.77	1.3	0.94	1.89	1.7	0.86	3.52

Satisfaction with low workload was significantly associated with both wellbeing and happiness (OR 2.8 95% CI 1.58-4.94 and OR 3.7 95% CI 1.94-7.09 respectively). Medium ideal work was also significantly associated with both wellbeing and happiness, while medium challenges were only associated with happiness.

A high workload was observed to be least associated with wellbeing and happiness. Ideal work and high workload were observed to be significantly associated with both wellbeing and happiness, while satisfaction was significantly associated with happiness regardless of workload. A medium workload, on the contrary, seemed to be most conducive to both wellbeing and happiness among seafarers onboard.

## Discussion

The main finding of this study is that there is a strong independent association between the work environment and the outcomes of wellbeing and happiness. Workload, however, showed to be an exception. Workload as a work environment factor is a subjective measurement, which should be seen in relation to the normal expected workload at sea. As seafarers have a relatively high workload while onboard, and the fact that they live at their workplace for months at end, we can assume that they find it difficult to leave or put aside work and the work environment completely at the end of the shift. The negative unadjusted association between workload and the outcome measures found in this study could be seen as a result of these circumstances.

It is interesting to note from the stratified multivariate regression analysis (see Table 6), that medium workload was significantly associated with both wellbeing and happiness. Moderation or having “just the right” amount to do, embodies the idea that wellbeing occurs when there is a balance between a person’s individual challenges and resources.^24^ Having too much or too little to do while onboard is not observed to be beneficial to the outcome of wellbeing and happiness. High job demands which are not in line with resources at hand, organizational or individual, may result in burnout, and reduced wellbeing and happiness.^19^

The positive associations between the outcome measures and most of the main exposure variables strongly suggest that the work environment has a great impact on the psychosocial wellbeing of seafarers currently at sea. Shea^40^ made a very thorough investigation on the onboard organizational culture and found that the team-based culture in practice 20+ years ago had an immense impact on the sense of wellbeing while at sea. It is noteworthy that within-industry reports today suggest that technological advancement is challenging the organizational culture promoting wellbeing onboard.^41^

This large-scale cross-sectional study can confirm the association found in previous, small-scale, studies about the association between the work environment on the one hand, and wellbeing and happiness on the other.^11,42^ Our findings also establish that the exposures to working conditions are independently associated with both wellbeing and happiness. This means that the link between the demands and procedures of onboard work and the feeling of happiness and wellbeing cannot be explained merely by the psychosocial dimensions experienced by the seafarers. This is important, as the onboard work processes is something that is affected by the tasks at hand and how they are organized by both the shipping company and the onboard staff, and not something governed by chance.^43,44^ Hence, the structure and process of work is something that can be managed in either better or worse ways.^45^

Drawing upon the definitions of wellbeing and happiness,^29,30^ the findings from within our study suggest that there is a relatively good balance between the different domains of wellbeing as defined by Labonté,^32^ but that this relationship needs nourishing to remain beneficial to the seafarers for example, physical health, mental wellbeing, social connections and support. This is also interesting from the perspective of how life at sea has changed with the introduction on onboard Internet access, where research reports have suggested that a decrease in social interaction during the free time onboard has a negative impact on the wellbeing of seafarers.^5,46,47^

Our study finds that there is especially a potential in further investigating the relationship between workload and the outcomes addressed in this study. In addition, it was interesting to find that the association between skills/training and the outcome measures was confounded by the psychosocial environment onboard. This suggests that the self-efficacy provided by training in relation to the tasks onboard, are affected by the interpersonal dynamics. It is worthwhile to point out that our analyses did not include measurements that have been suggested to affect the wellbeing of seafarers in other studies, such as how to deal with the separation from family,^48^ fear management,^40^ or personality traits.^33,34^

The maritime world has had a strong emphasis in the past couple of decades on safety. The recent interest in the wellbeing and happiness of seafarers is also related to the notion of safety. Namely, as there is a link between the prevalence of accidents and the wellbeing of seafarers. Seafarers that are happy with their current situation are also less prone to be involved in accidents.^49,50^ Hence, this study’s findings suggest that it is, from a safety perspective, good practice to safeguard a psychosocially healthy work environment as it would safety culture onboard and potentially reduce accidents.^3,12,36,51^

### Strengths and Limitations

The present study is an important contribution to the research literature on seafarers’ wellbeing and happiness in many ways. To start with, the population size in itself is rather unique. No other survey in the past has investigated this many seafarers at the same time and with the same instrument. In addition, the survey included seafarers from 154 different nationalities and—the proportion of these nationalities was representative for each of these nationalities. The same can be said of the proportion of officers and ratings, but also for age and gender.^1^

The sample size itself does not guarantee that it is representative for the global workforce of seafarers. However, in terms of gender composition (≈97% male), proportion of officers versus ratings (≈1/3 officers), and the proportion of seafarers from the main nations providing ship crews, ie, the Philippines (≈25%), India (≈10%), Russia (≈5%) and the Ukraine (≈5%) (see Table 2) the proportions within the sample matches those of the statistics available from established sources.^1^ Representativity itself is not based on a calculation, but rather on a comparison between the total population and the study sample given that the sample size is large, as is the case in the present study. There are of course other factors than the above that could determine the representativity of the present sample. However, the sample does indeed represent the hierarchical as well as national origin composition of the global seafaring community.

Nevertheless, there are limitations to the sampling. First, only 44 shipping companies volunteered to participate; even though these included some of the very largest companies in the world, many companies were missing.

Another important strength of this study was that it applied validated scales as measurements of the outcome variables. A common challenge in epidemiology is the vast range of different psychometric scales available to researchers. In this case, we decided to choose well-established validated measurements that could identify levels of wellbeing and happiness in a general population sample.

The major limitation of this study was the low response rate. Of the invited 160 000 seafarers, a little more than 10% completed the full survey. Online survey response rates, on land and presumably with a stable internet connection, normally vary between 20% and 30%.^52^ Internet provision and connectivity varied between different shipping companies. We did notice that some companies had significantly higher response rates than others. It could be that those seafarers that were not able to complete the full survey work in companies that do not sufficient internet or they work on substandard vessels. Hence, we can assume that this study is overrepresented by shipping companies that provide reliable internet onboard and invest in infrastructure onboard. Therefore, the responses might not be fully representative. In addition, the location of the vessel at the time of the survey could also contribute to the low internet connectivity, and thus the low response rate. We also observed that the response rate gradually reduced further into the survey. We can only speculate, but this could be due to the insufficient and slow internet connectivity, but also a loss of interest from the seafarers to complete the survey.

Another limitation was that a rather large number of participants initiated to reply to our questions but did not complete the questionnaire. As the respondents who chose to discontinue did so at different points in the survey a comprehensive non-respondent analysis was difficult to perform. However, there were no statistically significant differences in terms of age, sex, and current location among those who only finished the first part of the survey, where these questions were posed, and those who completed it in full. On the other hand, those who completed the survey had a statistically significantly higher probability of being officers and having spent shorter time onboard at their current posting. The answers from the ones who completed the questions regarding wellbeing and happiness scales but still did not complete the questionnaire, did not differ significantly from those who completed the full survey.

Another apparent limitation to this study was the cross-sectional design that did not allow us to draw any conclusions about inference between the psychosocial work environment and mental health outcomes. It could be assumed that someone who has a low level of wellbeing and/or happiness also experiences low satisfaction with their work situation, and vice versa. However, by including the psychosocial measurements in the multivariate regression analyses, this is to some extent controlled for.

## Conclusion

The present study found that there were independently significant associations between work related factors and wellbeing and happiness among a global sample of seafarers while working onboard at sea. The findings suggest that a greater emphasis on these outcomes could have a positive impact both on crew retention and safety at sea.

There are several recommendations for shipping companies to promote and improve wellbeing and happiness among their crew. Based on the findings from this study it is suggested to focus on work-related factors. Workload can be addressed by regular monitoring and assessments, to prevent stress and burnout. Duties onboard could be reorganized, and tasks evenly and fairly distributed among the crew. To alleviate the workload, it is also important for crew changes to take place in a timely manner, to implement reasonable rest hours, rest periods, rotation schedules, and increase crew members. Investing in training and skills development can enhance both the competence and confidence of seafarers, and thereby also ease the workload. It is also important that seafarers have balanced and correct expectations of work at sea. Make sure seafarers understand their roles and responsibilities and encourage them and to give feedback and share their concerns and experiences. Seafarers’ wellbeing and happiness impacts their work, safety, and satisfaction. By prioritizing these work-related factors, shipping companies can create a healthier and more beneficial work environment for their crew.
